# Preoperative stroke before cardiac surgery does not increase risk of postoperative stroke

**DOI:** 10.1038/s41598-021-88441-y

**Published:** 2021-04-27

**Authors:** Caleb R. Matthews, Timothy Hartman, Mackenzie Madison, Nicolas W. Villelli, Niharika Namburi, Cameron L. Colgate, Zainab Faiza, Lawrence S. Lee

**Affiliations:** 1 Division of Cardiothoracic Surgery, Indiana University School of Medicine, Indiana University Health Methodist Hospital, 1801 N. Senate Blvd., Suite 3300, Indianapolis, IN 46202 USA; 2 Department of Neurosurgery, Indiana University School of Medicine, Indianapolis, IN USA

**Keywords:** Outcomes research, Cardiology, Health care, Medical research, Risk factors

## Abstract

The optimal time when surgery can be safely performed after stroke is unknown. The purpose of this study was to investigate how cardiac surgery timing after stroke impacts postoperative outcomes between 2011–2017 were reviewed. Variables were extracted from the institutional Society of Thoracic Surgeons database, statewide patient registry, and medical records. Subjects were classified based upon presence of endocarditis and further grouped by timing of preoperative stroke relative to cardiac surgery: Recent (stroke within two weeks before surgery), Intermediate (between two and six weeks before), and Remote (greater than six weeks before). Postoperative outcomes were compared amongst groups. 157 patients were included: 54 in endocarditis and 103 in non-endocarditis, with 47 in Recent, 26 in Intermediate, and 84 in Remote. 30-day mortality and postoperative stroke rate were similar across the three subgroups for both endocarditis and non-endocarditis. Of patients with postoperative stroke, mortality was 30% (95% CI 4.6–66). Timing of cardiac surgery after stroke occurrence does not seem to affect postoperative stroke or mortality. If postoperative stroke does occur, subsequent stroke-related mortality is high.

## Introduction

Stroke in the perioperative period represents a major source of the stroke burden in the United States, with up to $4 billion in additional healthcare costs annually being attributed to cardiac surgery patients alone^1,2^. Compared to general surgical operations, cardiac surgery carries a much greater risk of stroke especially with valvular procedures^1–3^. Stroke following cardiac surgery has been shown to result in worse short- and long-term mortality as well as increased lengths of stay^3,4^.

Risk factors for postoperative stroke after cardiac surgery are numerous^1,3–5^. In fact, one such risk factor is previous stroke^3–6^. Timing of non-cardiac surgery has been well studied, and some have demonstrated elevated risk of postoperative stroke for up to nine months following neurological insult^7,8^. However, there is scant data regarding stroke and cardiac surgery, and the optimal timing when cardiac surgery can be safely performed after stroke is largely unknown. Guidelines from major cardiothoracic surgical professional societies generally indicate that surgery within 48 h of stroke is reasonable in the presence of strong surgical indications for surgery, but, if possible, to delay surgery for up to 4 weeks in cases of large territorial stroke or intracranial hemorrhage^9–12^. Some have shown acceptable outcomes for surgery within 2 weeks even in cases of intracranial hemorrhage^13,14^. There remains a paucity of data on whether the etiology of stroke plays a role; for example, stroke in patients with infective endocarditis (IE) is likely due to septic embolization, which may inherently be different than stroke in patients with atherosclerotic vascular and coronary artery disease.

We reviewed our experience with preoperative stroke in both IE and non-IE patient populations. We not only sought to define optimal timing from stroke to intervention but also to characterize how preoperative stroke impacts postoperative outcomes including stroke. We hypothesized that the shorter the duration between preoperative stroke and cardiac surgery, the greater the risk of postoperative stroke and mortality.

## Materials and methods

This single-center retrospective study was approved by the Institutional Review Board of Indiana University and conducted in accordance with all University guidelines and regulations. Informed consent by individual study patients was waived by the Institutional Review Board of Indiana University. We queried the prospective institutional Society of Thoracic Surgeons (STS) data registry to identify all patients with preoperative stroke who underwent cardiac surgery at our institution between 2011 and 2017 and also confirmed by ICD-9 codes. Cases where the index operation was ventricular assist device implantation, heart transplantation, or transcatheter valve replacement were excluded. Preoperative, intraoperative, and postoperative variables were extracted from this STS data registry, individual medical records, and a state-wide patient data registry (Regenstrief Institute, Indianapolis, IN). The latter allowed capture of patient data even if medical attention were received at an institution other than ours as long as it was within the state.

**Figure 1:**
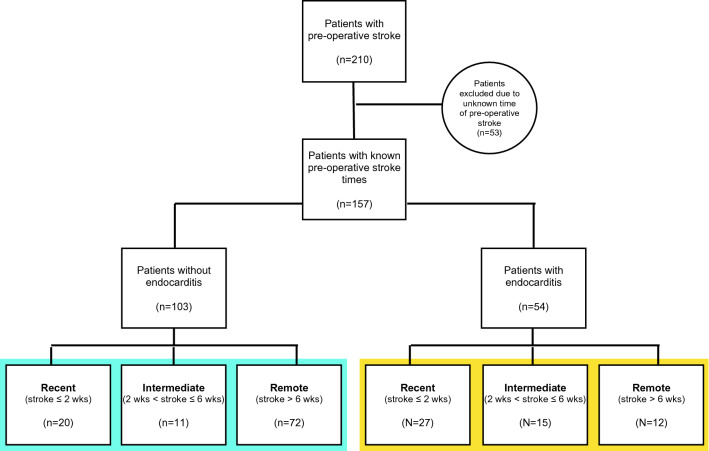
Diagram of study design.

Patients were divided into two groups based on the presence of IE (IE vs. non-IE), and then further classified by timing of the preoperative stroke relative to the cardiac surgical procedure date (Fig. 1): Recent (stroke within 2 weeks before surgery), Intermediate (greater than 2 weeks but less than 6 weeks before), and Remote (greater than 6 weeks before). Patients with unknown preoperative stroke date were excluded from analysis. All preoperative stroke imaging, either magnetic resonance imaging (MRI) or computed tomography (CT), was reviewed to ascertain anatomic location and territory. Postoperative outcomes were reviewed and compared amongst the three subgroups for both the IE and non-IE populations. Primary outcomes studied were 30-day mortality and incidence of postoperative stroke. Secondary outcomes included postoperative length of stay (LOS) and complications.

### Statistical analysis

Bivariate analysis was used to compare baseline characteristics between IE and non-IE groups. Kruskal–Wallis and Chi-Square tests were utilized for continuous and categorical variables, respectively. Using a backwards-stepwise logistic regression model, age, preoperative length LOS, and cross clamp time were selected as significant contributing variables for multivariable analysis against postoperative stroke rates. Boosted tree and random forest models also demonstrated these variables to be the top three important predictors of postoperative stroke. Statistical analysis was performed using RStudio (RStudio, Boston, MA) software.

## Results

210 patients were identified as having preoperative stroke prior to cardiac surgery. Of these, 50 were excluded from the study due to unknown timing of the preoperative stroke, leaving 157 subjects in the study: 54 in IE and 103 in non-IE. Within the IE group, 27 (50.0%) were in Recent, 15 (27.8%) in Intermediate, and 12 (22.2%) in Remote. In the non-IE group, 20 (19.4%) were in Recent, 11 (10.7%) in Intermediate, and 72 (69.9%) in Remote. Median follow-up in those who did and did not experience postoperative stroke are 1938 and 1070 days, respectively. Overall mortality was 6/157, or 3.8% (95% CI 1–7).

### Infective endocarditis

The mean age was 48 years, with 70% male and 69% Caucasian. Baseline characteristics were mostly similar across the three subgroups, with the exception of dyslipidemia and hypertension both being more common in the Remote group (Table 1). 37% had a history of illicit drug use. The majority of strokes were ischemic variant with either multi-territory or internal carotid artery anatomic distribution. The most common symptoms of the stroke were motor nerve defects and impaired consciousness. The proportion of cases classified as elective was different across the subgroups, comprising 41.7% of Remote cases and only 3.7% and 6.7% of Recent and Intermediate cases, respectively (Table 2). The majority of operations were isolated valve surgery with no significant differences in cardiopulmonary or crossclamp times across groups.

**Table 1:** Baseline demographics and pre-operative stroke-related characteristics by endocarditis cohort and stroke timing subgroup.

Characteristics	Non-endocarditis				Infective endocarditis			
	Recent (n = 20)	Intermediate (n = 11)	Remote (n = 72)	p-value	Recent (n = 27)	Intermediate (n = 15)	Remote (n = 12)	p-value
Age^a^ (years)	59.0 ± 13.6	54.9 ± 19.3	63.3 ± 14.6	0.209	48.1 ± 16.1	49.5 ± 15.9	48.8 ± 12.4	0.942
Body mass index^a^	29.4 ± 5.7	29.0 ± 7.0	28 ± 6.1	0.395	27.3 ± 7.3	27.7 ± 11.1	30.5 ± 7.9	0.433
Female	14 (70.0)	4 (36.4)	29 (38.9)	0.05	8 (29.6)	5 (33.3)	3 (25.0)	0.929
Non-white race	5 (25.0)	2 (18.2)	14 (19.4)	0.861	9 (33.3)	4 (26.7)	4 (33.3)	0.930
Smoker	2 (10.0)	4 (36.4)	7 (9.7)	0.086	7 (25.9)	2 (13.3)	3 (25.0)	> 0.999
Illicit drug use	2 (10.0)	3 (27.3)	5 (6.9)	0.082	11 (40.7)	4 (26.7)	5 (41.7)	0.482
Dyslipidemia	10 (50.0)	9 (81.8)	67 (93.1)	< 0.001	11 (40.7)	7 (46.7)	10 (83.3)	0.043
Renal failure	1 (5.0)	0	2 (2.8)	0.663	5 (18.5)	4 (26.7)	3 (25.0)	0.758
Diabetes	4 (20.0)	6 (54.5)	29 (40.3)	0.135	8 (29.6)	5 (33.3)	6 (50.0)	0.490
Chronic lung disease	4 (20.0)	4 (36.4)	27 (37.5)	0.383	6 (22.2)	2 (13.3)	2 (16.7)	0.899
Hypertension	16 (80.0)	8 (72.7)	67 (93.1)	0.043	10 (37)	8 (53.3)	10 (83.3)	0.031
Prior MI	1 (5.0)	3 (27.3)	33 (45.8)	0.001	2 (7.4)	5 (33.3)	3 (25.0)	0.080
**Stroke-related**								
Carotid Stenosis	3 (15.0)	1 (9.1)	10 (13.9)	0.908	2 (9.5)	2 (16.7)	1 (10)	0.834
History of TIA	1 (5.0)	2 (18.2)	6 (8.3)	0.545	1 (4)	0	1 (9.1)	0.468
Multiple prior strokes	2 (10.0)	1 (9.1)	3 (4.2)	0.292	0	0	0	N/A
*Stroke variant*				0.375				0.400
Ischemic	19 (95.0)	10 (90.9)	70 (97.2)		22 (81.5)	9 (60.0)	11 (91.7)	
Hemorrhagic	1 (5.0)	1 (9.1)	1 (1.4)		1 (3.8)	2 (13.3)	0 (0)	
Complex/Combined	0	0	1 (1.4)		4 (14.8)	4 (26.7)	1 (8.3)	
*Stroke location*				0.112				0.126
Diffuse	13 (65.0)	5 (45.5)	22 (30.6)		17 (63.0)	11 (73.3)	4 (33.3)	
ICA	4 (25.0)	4 (36.4)	30 (41.7)		7 (25.9)	3 (20.0)	8 (66.7)	
Vertebrobasilar	0	1 (9.1)	12 (16.7)		2 (7.4)	1 (6.7)	0	
Basal Ganglia	1 (5.0)	0	5 (6.9)		1 (3.7)	0	0	
Watershed	2 (10.0)	1 (9.1)	3 (4.2)		0	0	0	
*Symptoms*								
Ataxia	1 (5.0)	2 (18.2)	15 (20.8)	0.276	1 (3.7)	1 (6.7)	1 (8.3)	0.788
Motor nerve defect	14 (70.0)	4 (36.4)	36 (50.0)	0.152	8 (29.6)	5 (33.3)	7 (58.3)	0.236
Impaired consciousness	6 (30.0)	5 (45.5)	17 (23.6)	0.245	11 (40.7)	10 (66.7)	8 (66.7)	0.171
Cranial nerve deficit	4 (20.0)	4 (36.4)	34 (47.2)	0.092	3 (11.1)	3 (20.0)	3 (25)	0.511
Visual deficit	1 (5.0)	0	8 (11.1)	0.629	2 (7.4)	2 (13.3)	2 (16.7)	0.623
Seizures	1 (5.0)	2 (18.2)	4 (5.6)	0.224	1 (3.7)	0	0	> 0.999
Asymptomatic	1 (5.0)	0	3 (4.2)	1.00	6 (22.2)	2 (13.3)	0	0.215

Values are expressed as number (%) unless otherwise indicated.

*ICA* internal carotid artery, *MI* myocardial infarction, *TIA* transient ischemic attack.

^a^Mean ± standard deviation.

**Table 2:** Intraoperative details by endocarditis cohort and stroke timing subgroup.

Variable	Non-endocarditis				Infective endocarditis			
	Recent (n = 20)	Intermediate (n = 11)	Remote (n = 72)	p-value	Recent (n = 27)	Intermediate (n = 15)	Remote (n = 12)	p-value
Elective procedure	2 (10.0)	6 (54.5)	47 (65.3)	< 0.001	1 (3.7)	1 (6.7)	5 (41.7)	0.010
Procedure type				0.252				0.380
*CABG only*	2 (10.0)	2 (18.2)	24 (33.3)		0	0	0	
*Valve only*	1 (5.0)	1 (9.1)	13 (18.1)		15 (55.6)	6 (40.0)	8 (66.7)	
*CABG* + *Valve*	1 (5.0)	1 (9.1)	3 (4.2)		1 (3.7)	3 (20.0)	1 (8.3)	
*Other*	14 (70.0)	7 (63.6)	32 (44.4)		11 (40.7)	6 (40.0)	3 (25.0)	
CPB time^a^ (min)	143.8 ± 86.6	140.6 ± 110.4	150.3 ± 126.0	0.978	161.5 ± 74.8	180.7 ± 70.6	166.5 ± 96.8	0.570
Cross-clamp time^a^ (min)	78.0 ± 72.0	72.2 ± 102.0	72.0 ± 78.8	0.651	110.4 ± 52.7	140.0 ± 59.1	114.4 ± 54.0	0.401
IABP utilized	0	1 (9.1)	4 (5.6)	0.326	1 (3.7)	0	0	> 0.999
Preoperative length of stay (days)	5.5 (4.1)	8.0 (13.1)	2.2 (3.8)	0.002	7.9 (4.8)	17.9 (10.1)	8.2 (10.3)	0.002

Values are expressed as number (%) unless otherwise indicated.

*CABG* coronary artery bypass grafting, *CPB* cardiopulmonary bypass, *IABP* intra-aortic balloon pump.

^a^Mean ± standard deviation.

Postoperative stroke rates and 30-day mortality were 3.7% (95% CI 2–9) (n = 2) and 9.3% (95% CI 1.3–17) (n = 5), respectively, and were not different across the three subgroups (Table 3). Postoperative LOS was different across subgroups, with Remote having the shortest LOS (6.5 days) and Recent having the longest (13 days). Table 4 lists details of the postoperative strokes: patient 1 was in the Recent group with an ischemic stroke 7 days prior to undergoing mitral valve replacement with postoperative ECMO support. Postoperatively, stroke occurred on postoperative day 1 as an ischemic variant with multi-territory involvement and the patient died two days later due to multisystem organ failure. Patient 2 was in the Remote group with ischemic stroke 208 days prior to mitral valve replacement and suffered postoperative stroke on postoperative day 56 as a complex variant with left middle cerebral artery (MCA) territory involvement. There was hemorrhagic conversion of this stroke and the patient died 3 days later. There was no correlation between preoperative stroke variables, including variant, anatomic/territory involvement, and presenting symptom, and postoperative outcomes.

**Table 3:** Postoperative outcomes by endocarditis status and time cohort.

Postoperative outcome	Non-endocarditis				Infective endocarditis			
	Recent (n = 20)	Intermediate (n = 11)	Remote (n = 72)	p-value	Recent (n = 27)	Intermediate (n = 15)	Remote (n = 12)	p-value
Stroke	2 (10.0)	1 (9.1)	5 (6.9)	0.607	1 (3.7)	0	1 (8.3)	0.472
30-day mortality	1 (5.0)	1 (9.1)	3 (4.2)	0.605	3 (11.1)	1 (6.7)	1 (8.3)	> 0.999
Reoperation within 30 days	1 (5.0)	3 (27.3)	6 (8.3)	0.106	3 (11.1)	2 (13.3)	1 (8.3)	> 0.999
Atrial fibrillation^a^	7 (35.0)	1 (9.1)	8 (11.1)	0.039	5 (18.5)	1 (6.7)	2 (16.7)	0.609
Discharged home	6 (30.0)	5 (45.5)	49 (70.0)	0.003	9 (36)	4 (28.6)	8 (66.7)	0.122
Length of stay^b^, days	11.5 (6)	7.0 (21.5)	7.0 (4)	0.007	13 (21.5)	14.0 (12)	6.5 (5.0)	0.051

Reoperation for any reason within 30 days. Length of stay represents time from surgery to discharge. Values are expressed as number (%) unless otherwise indicated.

^a^Postoperative.

^b^Median and interquartile range.

**Table 4:** Details of study subjects who had postoperative stroke.

Patient	Pre-op stroke to surgery (days)	Pre-op stroke distribution	Pre-op stroke symptoms	Operation	Surgery status	Surgery to post-op stroke (days)	Post-op stroke distribution	Post-op stroke to stroke-related death (days)	Details
**Infective endocarditis**									
1	7	Diffuse	Hemiparesis; AMS	MVR, ECMO	Urgent	1	Diffuse	–	–
2	208	L Lateral- parietal	Dysarthria; AMS	MVR	Elective	56	L MCA	3	Hemorrhagic conversion
**Non-endocarditis**									
3	9	L Fronto- parietal	Hemiparesis; aphasia	Re-do MVR	Urgent	2	L MCA	–	–
4	13	L Thalamic	Hemiparesis	Re-do RVOT reconstruction	Elective	157	Diffuse	**–**	**–**
5	23	L MCA	Hemiparesis; dysarthria	CABG, AVR, VSD	Elective	0	Diffuse	–	–
6	1578	Diffuse	AMS	Aortic arch repair	Urgent	1	Posterior circulation	0	Tonsillar herniation
7	560	L ICA	Hemiparesis; dysarthria	CABG	Urgent	8	Occipital	–	–
8	1578	Diffuse	RUE paresis; amnesia	CABG	Urgent	1908	R Anterior Insular	–	–
9	154	R MCA, L Parietal	Hemiparesis; vision changes	CABG	Urgent	99	R Temporo-parietal	–	–
10	191	Diffuse	RUE paresis; dysarthria	TAAA repair	Elective	1	Diffuse	5	Brain death

*L* left, *R* right, *AMS* altered mental status, *MVR* mitral valve replacement, *ECMO* extracorporeal membrane oxygenation, *CABG* coronary artery bypass grafting, *AVR* aortic valve replacement, *RUE* right upper extremity, *ICA* internal carotid artery, *MCA* middle cerebral artery, *VSD* ventricular septal defect, *TAAA* thoracoabdominal aortic aneurysm.

### Non-endocarditis

Baseline demographics were similar across the three subgroups with respect to age and BMI. Prior MI, dyslipidemia, and hypertension were more common in the Remote group (Table 1). The majority of pre-operative strokes were ischemic and symptomatic with motor nerve involvement, but there was no difference amongst the three subgroups. There were significantly more elective cases in the Remote group than others and the types of cases across groups were similar. Intraoperative variables were similar, including the duration of cardiopulmonary bypass and crossclamp period (Table 2).

Postoperative stroke rate and 30-day mortality were 7.7% (n = 8) (95% CI 2.5–13) and 4.9% (n = 5) (95% CI 7–9.4), respectively, and were not different across subgroups (Table 3). Remote had the lowest postoperative stroke rate followed by Intermediate and then Recent, although this was not statistically significant. Postoperative atrial fibrillation (AF) was most common in Recent, followed by Intermediate and then Remote. Postoperative LOS was shortest in the Remote group (7.0 days) and longest in Recent (11.5 days), with Remote having the greatest proportion that discharged to home (vs. an institutional facility).

All postoperative strokes were ischemic in nature and the occurrence of stroke ranged from day of surgery to postoperative day 1908 (Table 4), with most occurring within the first 10 days after surgery. Of the patients who suffered postoperative stroke, three died due to stroke related complications, including hemorrhagic conversion, tonsillar herniation, and absence of neurologic recovery/brain death. These stroke deaths occurred relatively quickly after stroke, ranging from day of stroke to 5 days after stroke. Multivariate logistic analysis did not identify any particular variables that predicted postoperative stroke in any of the subgroups (Table 5).

**Table 5:** Multivariable logistic regression analysis of variables selected based upon results of backwards stepwise regression, boosted tree model, and random forest model.

Variable	Non-endocarditis		Infective endocarditis	
	Odds ratio [95% CI]	p-value	Odds ratio [95% CI]	p-value
(Intercept)	0.01 [0.00, 0.49]	0.021	0.00 [0.00, 0.87]	0.028
Recent (compared to remote)	1.31 [0.25, 6.93]	0.752	1.21 [0.07, 20.83]	0.897
Intermediate (compared to remote)	1.13 [0.13, 9.95]	0.914	0.12 [0.00, 3.14]	0.208
Age	1.03 [0.98, 1.08]	0.304	1.02 [0.94, 1.12]	0.601
Preoperative length of stay	1.06 [0.95, 1.19]	0.287	1.12 [0.98, 1.28]	0.105
Cross clamp time	1.05 [0.61, 1.83]	0.849	2.30 [0.53, 9.92]	0.267

*CI* confidence interval.

## Discussion

There remains little published data regarding the effect of preoperative stroke and outcomes following cardiac surgery. Most recommendations are based on either the experience of non-cardiac surgery or observational studies focusing on the IE population in cardiac surgery^8,13–18^. The decision and timing of cardiac surgery following stroke remains debated. Our results suggest that there is no direct relationship between timing of cardiac surgery after a stroke and postoperative outcomes of stroke and mortality—our data do not reveal a protective effect of time between stroke and cardiac surgery. Time to intervention from preoperative stroke did not influence frequency of mortality or postoperative stroke in either IE or non-IE populations. This contradicts some published data that suggest there is an increased risk of postoperative stroke particularly in IE, and that waiting 2–4 weeks following stroke might reduce the risk of postoperative stroke^8–12,19^. However, other investigators have reported results similar to ours, whereby preoperative stroke in IE is not associated with significant increase in postoperative stroke after valve surgery^14,20,21^. We surmise that preoperative stroke in IE is primarily related to septic emboli, and it is likely that induction of antimicrobial therapy prior to cardiac surgery reduces subsequent stroke risk^22^. Unlike the non-IE groups, there is likely no underlying atherosclerotic disease in patients with IE that would predispose to stroke occurrence. Based on our data, in patients with IE it appears that the presence of stroke should not necessarily affect timing of surgery, as there is no significant increase in postoperative mortality or stroke.

In the non-IE population, our data show a greater rate of postoperative stroke (7.7%) than in IE, but again there does not seem to be a correlation with the time interval between preoperative stroke and surgery. The Recent group had a greater absolute percentage of postoperative stroke than the Remote group, but this difference was not statistically significant. Interestingly, the rates of postoperative AF were greatest in Recent and lowest in Remote, which initially led us to believe that postoperative AF and postoperative stroke were related. However, we could not find a meaningful association between the two. Among the cases with postoperative stroke, there were equal numbers of isolated coronary bypass and isolated valve surgery. The strokes following isolated coronary surgery seemed to occur later, but again this was not statistically significant. The territory and symptoms of postoperative stroke were not affected by details of the preoperative stroke; in other words, a patient with hemiparesis due to left middle cerebral artery involvement in preoperative stroke did not place the patient at higher risk for a similarly located postoperative stroke. Furthermore, six of the ten postoperative occurred within the first ten postoperative days; the other four occurred at postoperative day 56, 99, 108, and 1908. We surmise that the six strokes occurring in the early postoperative period are likely related to the surgery itself (as a postoperative complication), while the four that occurred more than one month after surgery are likely unrelated to surgery. Even taking into account these late strokes, our data suggest that the presence of pre-operative stroke does not definitively increase risk of postoperative stroke. As with IE, in non-IE patients with a history of stroke, our data suggest that cardiac surgery can be performed without affecting postoperative mortality or stroke, regardless of interval between stroke and surgery.

Across all patients who have postoperative stroke, the stroke-related mortality was 30%. This is consistent with a recent review by Sultan et al. of 10,250 patients which revealed a mortality of 14.9% in patients who suffered a symptomatic postoperative stroke after cardiac surgery^23^. This underscores the importance of preventing postoperative stroke, as it seems the presence of postoperative stroke correlates with increased mortality. Before discharge all patients are administered medical therapy for cardiovascular optimization (blood pressure control, antiplatelets/anticoagulation, hyperlipidemia control, etc.) as long as there are no contraindications. The specific agents were determined often by preoperative baseline medications (if a patient was on amlodipine preoperatively, for example, that agent was resumed) and contemporary cardiac surgery/cardiology guidelines (for instance, antiplatelets with either aspirin or clopidogrel as well as a statin for patients undergoing coronary bypass surgery). It is possible that the variability in postoperative medical regimen could have affected risk of postoperative stroke, but the present study was not designed to distinguish this effect. Nonetheless, the timing of surgery following preoperative stroke does not appear to affect postoperative stroke rate and our data would not support delaying cardiac surgical intervention merely due to the presence of stroke alone.

There are limitations to this single center retrospective study. First, the relatively low sample size combined with the low incidence of postoperative stroke and mortality may contribute to difficulty in identifying significant relationships. We had initially sought to utilize a larger multi-institutional data registry, yet there was no such registry that contained the granular level of information (e.g., stroke variant, territory involvement, symptoms, intraoperative details, postoperative stroke and death details, etc.) necessary to test our hypothesis. Analyzing individual patient level data allowed us to investigate more detailed variables than would have been possible with larger registries. Second, this study selected for patients who ultimately underwent cardiac surgery. Excluded were patients who were too ill from the preoperative stroke to tolerate cardiac surgery, which may have affected outcomes. Our institutional database does not allow for identification of patients who were deemed not surgical candidates and were treated with non-surgical modalities for their cardiac disease.

## Conclusions

Our study suggests that the presence of preoperative stroke may not affect postoperative mortality and stroke, and that cardiac surgery can be safely performed regardless of the time interval between stroke and surgery. Preoperative variables including details of preoperative stroke do not correlate with risk of postoperative stroke. Multiple factors are considered in the decision and timing of cardiac surgery, but the presence and type of preoperative stroke may not need to play a major role.
